# Elevated expression of STIM1 is involved in lung tumorigenesis

**DOI:** 10.18632/oncotarget.13359

**Published:** 2016-11-15

**Authors:** Yadong Wang, Haiyu Wang, Li Li, Jiangmin Li, Teng Pan, Ding Zhang, Haiyan Yang

**Affiliations:** ^1^ Department of Toxicology, Henan Center for Disease Control and Prevention, Zhengzhou 450016, China; ^2^ Henan Collaborative Innovation Center of Molecular Diagnosis and Laboratory Medicine, Xinxiang Medical University, Xinxiang 453003, China; ^3^ Department of Epidemiology, School of Public Health, Zhengzhou University, Zhengzhou 450001, China

**Keywords:** STIM1, A549 cell, lung cancer, tumorigenicity, cell cycle

## Abstract

This study aimed to address the potential role of STIM1 (stromal interaction molecule 1) in lung tumorigenesis. Colony formation in soft agar assay and tumorigenicity in nude mice assay were conducted. Western blot, immunohistochemistry and quantitative real-time polymerase chain reaction were used to measure the STIM1 expression. The distribution of cell cycle was detected by flow cytometry assay. Our results showed that the expression of STIM1 mRNA was significantly higher in human lung tumors than that in adjacent non-neoplastic lung tissues. Significantly increased expression of STIM1 mRNA and protein was observed in 16HBE-benzo(a)pyrene (BaP) cells and in BaP-treated mice lung tissues compared with 16HBE-control cells and the control group, respectively. Silencing STIM1 inhibited the proliferation and colony formation of A549 cells in *in vitro* experiments, attenuated the growth of tumor xenografts of A549 cells in *in vivo* experiments and induced the arrest of cell cycle in the G1 phase. The markedly decreased expression of cyclin D1 protein was observed in A549-shRNA-STIM1 cells as compared to A549-shRNA-control cells. The markedly increased expression of p21 protein was observed in A549-shRNA-STIM1 cells as compared to A549-shRNA-control cells. The expression levels of β-catenin and TGIF proteins were lower in A549-shRNA-STIM1 cells than those in A549-shRNA-control cells. In conclusion, this study indicated that the elevated expression of STIM1 might be involved in lung tumorigenesis.

## INTRODUCTION

An estimated 1.8 million new lung cancer cases occurred in 2012, accounting for approximately 13% of total cancer diagnoses. In men, lung cancer was the most commonly diagnosed cancer and the leading cause of cancer-related death in 2012. In women, lung cancer was the leading cause of cancer-related death in more developed countries and the second leading cause of cancer-related death in less developed countries [1]. Smoking, particularly of cigarettes, is by far the main contributor to lung cancer, and other risk factors include exposure to radon gas, asbestos, air pollution and some metals, and genetic alterations [2–8]. Similar to many other cancers, lung cancer is initiated by activation of oncogenes or inactivation of tumor suppressor genes [9–11]. The molecular mechanisms involved in lung tumorigenesis remain incompletely understood.

STIM1 (stromal interaction molecule 1) was identified as a novel human gene that maps to a region of chromosome 11p15.5 [12]. STIM1 protein is mainly resident in the endoplasmic reticulum (ER), and activates a set of plasma membrane Ca^2+^ channels termed store-operated calcium channels (SOCs) when the concentration of free Ca^2+^ within the ER drops transiently as a result of Ca^2+^ release from this compartment [13]. It has been reported that STIM1 protein has novel and unexpected physiological and pathophysiological roles in several tissues [14], the clinical phenotype of STIM1-deficient patients was characterized by immunodeficiency together with autoimmune disease, congenital myopathy and ectodermal dysplasia [15].

STIM1 was initially suggested to be a tumor suppressor given that it induced the inhibition of rhabdoid tumor and rhabdomyosarcoma cell lines [16]. Suyama et al. reported that knocking down STIM1 accelerated the cell motility of melanoma cells, and STIM1 was identified as an anti-metastasis gene [17]. However, recent data reveal opposite functions. Wong et al. reported that the presence of STIM1 overexpression in colon adenocarcinomas was associated with cell migration and cell motility properties [18]. Yang et al. reported that STIM1 silencing inhibited the migration and metastasis of breast cancer cells [19]. Chen et al. reported that a poorer clinical outcome was statistically significant in primary tumors with STIM1 upregulation. STIM1 overexpression markedly enhanced cervical tumor cell growth, local spread and angiogenesis and promoted cancer cell migration and invasion [20]. Additional studies showed that knocking down STIM1 resulted in reduced cancer metastasis in colorectal cancer [21], melanoma [22, 23] and hepatocellular carcinoma [24].

However, the potential role of STIM1 in lung tumorigenesis has not yet been clearly understood. One published paper reported that higher levels of STIM1 protein expression were observed in the samples of patients with non-small cell lung cancer (NSCLC) compared with adjacent non-neoplastic lung samples as detected by immunoblotting analysis and immunohistochemistry [25]. To further understand the potential role of STIM1 in lung tumorigenesis, we measured the expression levels of STIM1 in malignant human bronchial epithelial (16HBE) cells transformed by benzo(a)pyrene (BaP) and mice lung tissues treated with BaP. We also investigated the influences of STIM1 silencing on the proliferation and tumor formation of A549 cells in *in vitro* and *in vivo* experiments, and the distribution of cell cycle of A549 cells.

## RESULTS

### Elevated STIM1 expression in human lung tumors

**Figure 1:**
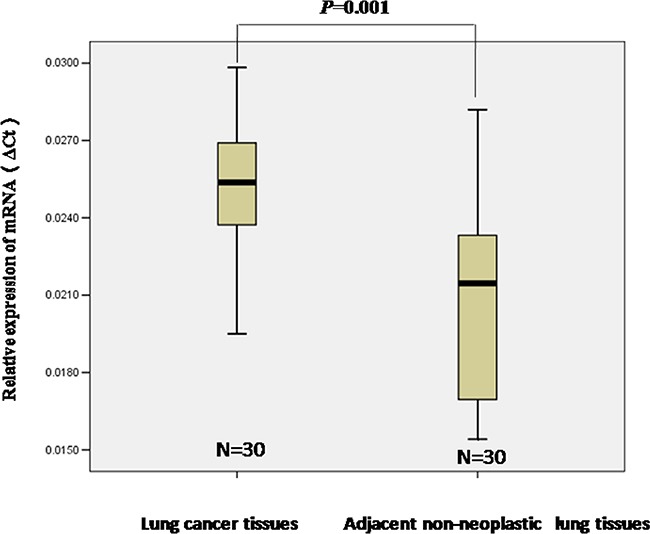
The relative levels of STIM1 mRNA expression was detected by qRT-PCR The level of STIM1 mRNA expression was higher in lung tumors than that in adjacent non-neoplastic lung tissues. Median values are indicated, and the Mann-Whitney U test was performed.

To investigate whether or not STIM1 has a potential role in lung cancer, we examined STIM1 expression by qRT-PCR assay. Thirty human lung cancer tissues and paired adjacent non-neoplastic lung tissues were collected. Our results demonstrated that STIM1 mRNA expression was higher in human lung tumors than that in adjacent non-neoplastic lung tissues (*P* = 0.001) (Figure 1).

### Elevated STIM1 expression in malignant 16HBE cells transformed by BaP and mice lung tissues treated with BaP

**Figure 2:**
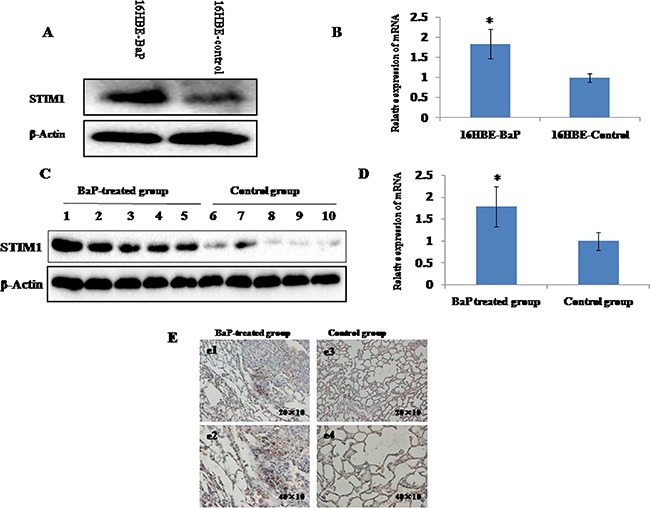
STIM1 expression in malignant 16HBE cells transformed by BaP and BaP-treated mice lung tissues were measured by qRT-PCR and western blot assay The levels of STIM1 protein **A**. and mRNA **B**. were higher in malignant 16HBE cells transformed by BaP than those in control cells. The levels of STIM1 protein **C**. and mRNA **D**. were higher in BaP-treated mice lung tissues than those in the control group. The level of STIM1 in BaP-treated mice lung tissues was detected by immunohistochemistry **E**. Plates e1 (200×) and e2 (400×) represent the lung sections of BaP-treated group, and plates e3 (200×) and e4 (400×) represent the lung sections of the control group. The BaP-treated group exhibited strong cytoplasmic and nuclear STIM1 staining in some cells (e1 and e2). * *P* < 0.05.

STIM1 mRNA and protein expression profiles were examined by qRT-PCR and western blot assay. Increased STIM1 protein (Figure 2A) and mRNA (Figure 2B) expression was observed in 16HBE-BaP cells compared with 16HBE-control cells. Consistent with the data from cell line, increased expression of STIM1 protein (Figure 2C) and mRNA (Figure 2D) was observed in mice lung tissues treated with BaP as compared with the control group. The immunohistochemistry analysis demonstrated that the staining signal of STIM1 was strong in the nucleus and cytoplasm in some cells in the BaP-treated group but weak in the control group (Figure 2E).

### Silencing efficiency of STIM1 gene in A549 cells

**Figure 3:**
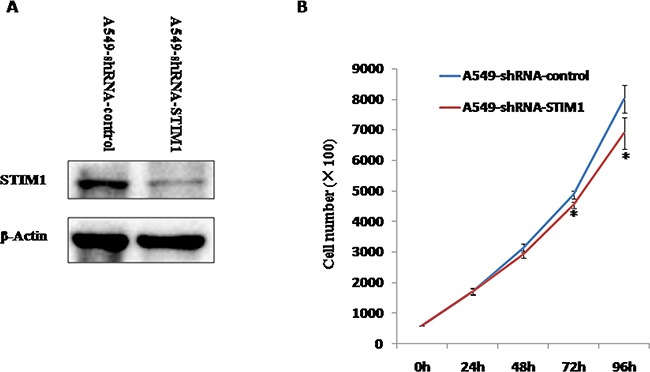
The silencing efficiency of STIM1 in A549 cells and the effects of silencing STIM1 on A549 cell proliferation **A**. Western blot analysis showed that shRNA specifically targeting STIM1 markedly decreased the level of STIM1 protein expression in A549 cells compared with control. **B**. STIM1 silencing inhibited A549 cell proliferation at 72 h and 96 h. *P<0.05.

Figure 3A showed that the markedly decreased expression of STIM1 protein was observed in A549-shRNA-STIM1 cells as compared to A549-shRNA-control cells. This observation suggested that an A549 cell line stably silencing STIM1 was successfully engineered.

### Silencing STIM1 suppressed the proliferation of A549 cells

We investigated the effects of STIM1 silencing on the proliferation of A549 cells. Our results indicated that the significantly slower growth rate was observed in A549-shRNA-STIM1 cells as compared to A549-shRNA-control cells at 72 h and 96 h (*P* < 0.05).

### Silencing STIM1 attenuated the colony formation in soft agar of A549 cells

**Figure 4:**
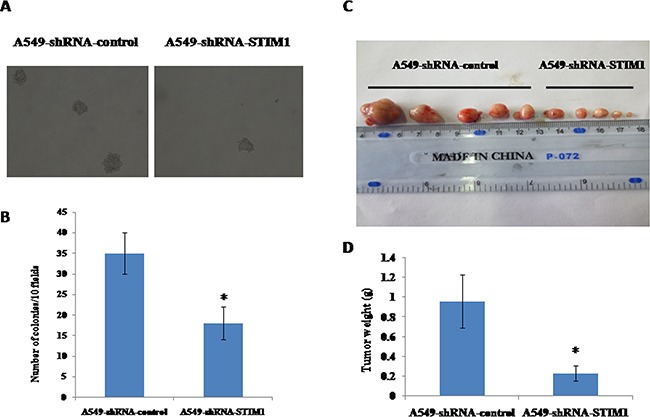
Effects of silencing STIM1 on colony formation and tumor growth in mouse xenograft models Dramatically decreased colony formation was observed in A549-shRNA-STIM1 cells compared with A549-shRNA-control cells **A** and **B**. The mean of tumor weight was significantly reduced in A549-shRNA-STIM1 cells compared with A549-shRNA-control cells **C** and **D**. *P<0.05.

After observing that silencing STIM1 inhibited A549 cell proliferation, we further investigated whether or not silencing STIM1 had effects on the colony formation in soft agar of A549 cells *in vitro*. As shown in Figure 4A and 4B, A549-shRNA-STIM1 cells (18±4) exhibited markedly decreased colony formation compared with A549-shRNA-control cells (35±5) (*P* < 0.05).

### Silencing STIM1 suppressed tumor growth of A549 cells in vivo

We observed that silencing STIM1 inhibited colony formation in soft agar of A549 cells *in vitro*. To test the potential role of silencing STIM1 in the ability of tumor growth of A549 cells *in vivo*, nude mice xenograft models were established by subcutaneous injection with A549-shRNA-STIM1 cells and A549-shRNA-control cells. As shown in Figure 4C and 4D, on the 16^th^ day, the mean of tumor weight was significantly decreased in the group of mice injected with A549-shRNA-STIM1 cells (0.23±0.08 g) as compared to the group of those injected with A549-shRNA-control cells (0.96±0.27 g) (*P*<0.05), suggesting that silencing STIM1 markedly suppressed the growth and formation of tumors of A549 cells in *in vivo* experiments.

### Silencing STIM1 arrested cell cycle in G1 phase

**Figure 5:**
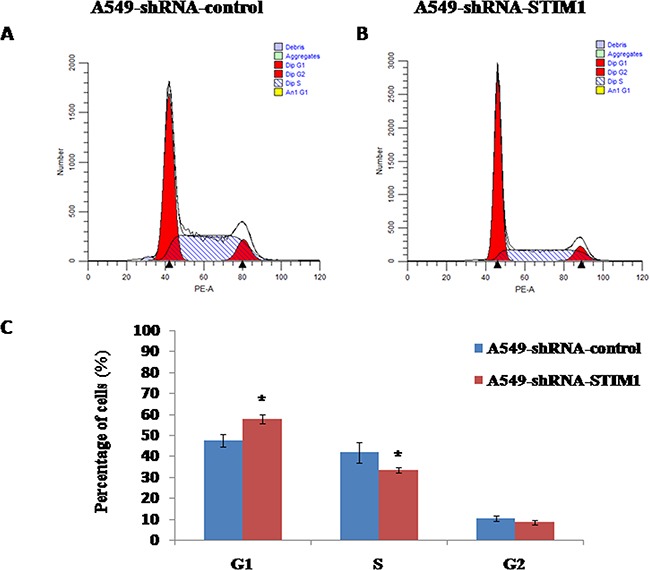
Effects of STIM1 silencing on cell cycle distribution Representative flow cytometric histograms showed the distribution of cell cycle in A549-shRNA-control cells **A**. and A549-shRNA-STIM1 cells **B**. STIM1 silencing increased the percentage of cells in G1 phase and decreased the percentage of cells in S phase **C**. *P<0.05.

We observed that silencing STIM1 inhibited A549 cell proliferation. To further investigate whether or not the cell growth inhibition was induced by cell cycle arrest, we performed the flow cytometry assay to detect the distribution of cell cycle. Our results showed that the percentage of G1 phase cells was significantly higher in A549-shRNA-STIM1 cells (57.9±2.1%) than that in A549-shRNA-control cells (47.7±2.9%) (*P*<0.05), and the percentage of S phase cells was markedly decreased in A549-shRNA-STIM1 cells (33.4±1.2%) than that in A549-shRNA-control cells (41.9±4.9%) (*P*<0.05) (Figure 5). In addition, we observed that the proliferation index in A549-shRNA-STIM1 cells (42.0±2.2%) was markedly decreased as compared to that in A549-shRNA-control cells (52.3±2.9%) (*P*<0.05). These findings suggested that silencing STIM1 might inhibit the growth of A549 cells by arresting the cell cycle in the G1 phase.

### Silencing STIM1 downregulated cyclin D1 expression and upregulated p21 expression

**Figure 6:**
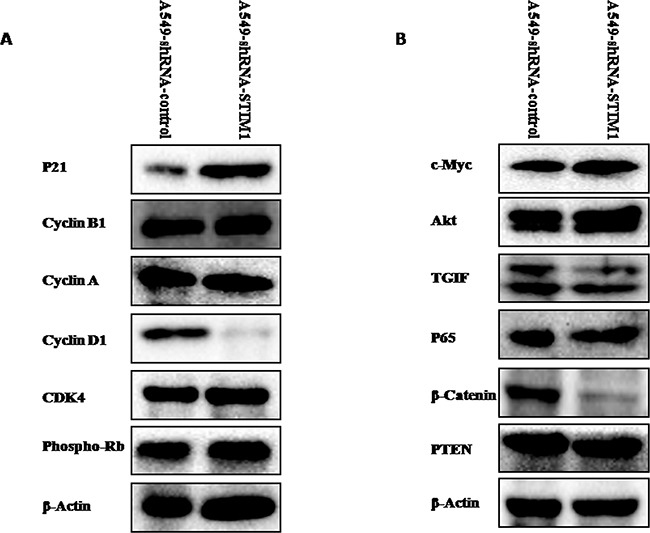
Effects of silencing STIM1 on the expression of several proteins Western blot analysis showed that silencing STIM1 decreased cyclin D1, TGIF and β-catenin expression and increased p21 expression.

To explore the potential mechanism related to the cell cycle arrest, the expression levels of cell cycle-related proteins were measured by western blot analysis. Our results indicated that silencing STIM1 reduced cyclin D1 protein expression and increased p21 protein expression (Figure 6A). No significant alterations in the expression levels of cyclin B1, cyclin A, CDK4 and phospho-Rb proteins were noted between A549-shRNA-STIM1 cells and A549-shRNA-control cells (Figure 6A).

### Silencing STIM1 downregulated the expression levels of β-catenin and TGIF proteins

We further tentatively investigated whether or not silencing STIM1 could have effects on the expression levels of Akt, c-Myc, TGIF, β-catenin, p65 and PTEN proteins, since these proteins play important roles in lung tumorigenesis. As shown in Figure 6B, western blot results indicated that the expression levels of β-catenin and TGIF proteins were significantly reduced in A549-shRNA-STIM1 cells as compared to A549-shRNA-control cells. No significant alterations in the expression levels of PTEN, c-Myc, p65 and Akt proteins were noted between A549-shRNA-STIM1 cells and A549-shRNA-control cells.

## DISCUSSION

In this present study, higher expression of STIM1 mRNA was observed in lung tumors compared with adjacent non-neoplastic lung tissues, suggesting that STIM1 overexpression may be involved in lung tumorigenesis. A previous study observed higher STIM1 protein expression in lung tumors compared with adjacent non-neoplastic lung tissues detected by immunoblotting analysis and immunohistochemistry [25]. Our results may enhance the confidence of previous findings to a certain extent. To confirm the results observed in human samples, we measured STIM1 mRNA and protein expression in malignant human bronchial epithelial cells transformed by BaP and lung tissues from mice treated with BaP. Our results consistently showed that higher expression of STIM1 mRNA and protein was observed in malignant 16HBE cells transformed by BaP and lung tissues from mice treated with BaP compared with corresponding controls, which supported our findings at the cellular level and in an animal model to a certain extent.

Previous experimental studies showed that suppression of STIM1 inhibited cell proliferation of glioblastoma [26], melanoma [22], hepatocellular carcinoma [27], human epidermoid carcinoma [28] and hypopharyngeal carcinoma [29]. In this current study, we observed that silencing STIM1 inhibited A549 cell proliferation. Combined with previous results, silencing STIM1 inhibited the growth of many types of tumor cells. To further investigate the effects of silencing STIM1 on tumor formation *in vitro* and *in vivo*, a soft agar colony formation assay was performed, and a mouse tumor xenograft model was established. Our results demonstrated that suppression of STIM1 reduced the tumor growth and formation of human non-small cell lung cancer cell line of A549 in *in vitro* experiments and in *in vivo* experiments, which confirmed our above observations that STIM1 might play an important role in lung tumorigenesis.

One of the major causes of reducing the proliferation of cancer cells is cell cycle arrest. Our data indicated that silencing STIM1 arrested the cell cycle of A549 in G1 phase. The significantly increased percentage of cells in G1 phase was observed in A549-shRNA-STIM1 cells as compared to that in A549-shRNA-control cells. Accompanied with G1 phase arrest, we observed the downregulation of cyclin D1 protein in A549-shRNA-STIM1 cells. Studies showed that increased cyclin D1 expression in cancer cells resulted in an uncontrolled growth advantage [30]. In addition, silencing STIM1 in this study significantly increased p21 protein expression. p21 is one of the major cyclin-dependent kinase inhibitors (CDKI), which exerts negative effects on cell cycle progression and arrests proliferation by binding to active cyclin-CDK (cyclin-dependent kinase) complex and inhibiting their kinase activities [31–34]. Lin et al. observed that STIM1 silencing in U251 cells resulted in a marked decrease in cyclin D1 expression and a marked increase in p21 expression [26]. Chen et al. reported that knocking down STIM1 induced the expression of p21 in cervical cancer SiHa cells [20]. Taken together, the silencing of STIM1 induced G1 phase cell cycle arrest may be mediated via downregulating cyclin D1 and upregulating p21 in A549 cells. However, the potential molecular mechanisms of STIM1 regulating the expression of cyclin D1 and p21 were not investigated in this present study and should be the focus of future studies. A previous study proposed that posttranslational regulation via the 26S-proteasome-dependent pathway might contribute to the mechanisms controlling p21 upregulation in STIM1-knock-down cervical cancer cells [20].

TGIF (TG-interacting factor) was initially reported as a negative regulator of retinoic acid (RA) and the transforming growth factor β (TGF-β) signaling pathway [35, 36]. Zhang et al. reported that TGIF participated in the regulation of Wnt/β-catenin signaling pathway [37]. Our published paper revealed that the increased expression of TGIF was related to lung tumorigenesis [38]. Silencing TGIF suppressed the growth and tumor formation of A549 cells [39]. In this current study, silencing STIM1 suppressed the expression of TGIF and β-catenin proteins. One published paper reported that TGIF could regulate the expression of β-catenin protein in non-small cell lung cancer cells. Overexpression of TGIF increased the expression of β-catenin protein, and knocking down TGIF reduced the expression of β-catenin in A549 cells [40]. Taking these findings together, STIM1 may play a potential role in regulating Wnt/β-catenin signaling in NSCLC cells.

In summary, our data suggest that increased expression of STIM1 has a potential role in lung tumorigenesis. Silencing STIM1 attenuated the tumor growth of A549 cells. In addition, STIM1 suppression induced A549 cell cycle arrest in G1 phase by upregulating p21 and downregulating cyclin D1. This study provides new insights into the molecular oncogenesis of lung cancer and suggests that STIM1 may be a potential molecular target for human lung cancer therapy.

## MATERIALS AND METHODS

### Cell lines

The A549 cell line was cultured in RPMI-1640 medium according to our published paper [39]. Control shRNA lentiviral particles-A (sc-108080) and STIM1 shRNA (h) lentiviral particles (sc-76589-V) were obtained from Santa Cruz Biotechnology (Santa Cruz, CA, USA). We infected A549 cells with lentiviral particles according to the manufacturer's instructions (Santa Cruz, CA, USA). The infected A549 cells were cultured in RPMI 1640 with 10 μg/ml of puromycin for 3 weeks (Gibco, USA). The stable clone infected with STIM1 shRNA (h) lentiviral particles was termed A549-shRNA-STIM1 and the stable clone infected with control shRNA lentiviral particles was termed A549-shRNA-control. The malignant 16HBE cell line transformed by BaP (16HBE-BaP) and its control cell line (16HBE-control) were maintained in our laboratory [38].

### Patient samples

Samples from thirty human lung cancer patients were reported in our previous study [38]. The histological diagnosis of the specific type of lung cancer was determined according to the recommendations of the WHO. The Ethics Committee of the First Affiliated Hospital of Zhengzhou University approved this work, and we carried out the experiments in accordance with the relevant guidelines, including any relevant details. All patients participating in this study provided informed consent.

### Cell proliferation analysis

Cells (A549-shRNA-STIM1 and A549-shRNA-control) were seeded in 12-well plates (4×10^4^ cells/well). At 24, 48, 72 and 96 h after seeding, cells were detached and counted in accordance with our previous paper [39].

### Colony formation in soft agar assay

The detailed procedures of soft agar colony formation assay were described in our previous publications [38, 39].

### Nude mice tumorigenicity

The Ethics Committee of Henan Center for Disease Control and Prevention approved this work. All animal experiments were carried out in accordance with the approved guidelines. Ten female BALB/c nude mice aged four weeks were obtained from Vital River Laboratory Animal Technology Co. Ltd (Beijing, China). Cells (A549-shRNA-STIM1 and A549-shRNA-control) were prepared and injected to each mouse in accordance with our previous papers [38, 39]. The mice were sacrificed at 16 days post-injection.

### Quantitative real-time polymerase chain reaction (qRT-PCR)

Total RNA was extracted using RNAiso Plus (Takara, Japan). cDNA was synthesized using the reverse transcriptase kit (Prime ScriptH RT reagent Kit-Perfect Real Time, Takara, Japan). The cDNA products were analyzed using the SYBR Green PCR Master Mix (Premix Ex Taq^TM^-Perfect Real Time, Takara, Japan) and 7300 Real-Time PCR machine (Applied Biosystems) according to the manufacturer's instructions. The primers sequences used were as follows: mouse STIM1, 5’-TGAAGAGTCTACCGAAGCAGA-3’ (sense) and 5’-AGGTGCTATGTTTCACTGTTGG-3’ (antisense); human STIM1, 5’- CCTCGGTACCATCCA TGTTGTAGCA -3’ (sense) and 5’- GCGAAAGC TTACGCTAAAATGGTGTCT-3’ (antisense); and GAPDH, 5’- GACCCCTTCATTGACCTCAAC-3’ (sense) and 5’-CTTCTCCATGGTGGTGAAGA-3’(antisense). Relative STIM1 gene expression was represented as 2^-ΔCt^ (ΔCt=Ct_STIM1_-Ct_GAPDH_).

### Western blot assay

Tissues or cells were lysed in RIPA buffer (Pierce, USA) supplemented with phosphatase inhibitors and protease inhibitors (Pierce, USA), and the protein concentration was quantified by the BCA kit (Pierce, USA). Soluble lysates (30 μg protein) were fractionated by 10% SDS-PAGE gels and transferred to nitrocellulose (NC) membranes (PALL, USA). The membranes were immersed in Tris-buffered saline-Tween 20 (TBST) containing 5% bovine serum albumin (BSA) to block non-specific background staining and then incubated at 4°C overnight with primary antibody. STIM1 (sc-68897), TGIF (sc-9084), Akt (sc-8312), cyclin A (sc-751), cyclin B1 (sc-752), CDK4 (sc-260), cyclin D1 (sc-718), p21 (sc-397), and β-Actin (sc-8432) were obtained from Santa Cruz Biotechnology (Santa Cruz, CA, USA). PTEN (#9188S), phospho-Rb (#8516S), p65 (#8242S), β-catenin (#9582S), and c-Myc (#13987S) were obtained from Cell Signaling Technology (Cell Signaling, USA). The membranes were washed with TBST and incubated with goat anti-rabbit or mouse-IgG secondary antibodies (peroxidase conjugated, ZSGB-BIO, Beijing, China) at room temperature for 1 h. The immunoreactive blots were developed [39].

### Cell cycle assay

The flow cytometry (BD, Biosciences) was applied to measure the distribution of cell cycle. The detailed procedures of cell cycle analysis were reported in our previous paper [39].

### STIM1 immunohistochemistry

BaP-treated mice lung tissues and control lung tissues were maintained in our laboratory [38]. The Ethics Committee of Henan Center for Disease Control and Prevention approved this work. All the animal experiments were performed in accordance with the approved guidelines. Immunohistochemistry analysis was performed according to the manufacturer's instructions (Beijing ComWin Biotech Co., Ltd; CW0120).

### Statistics

The data are presented as the mean ± standard deviation (SD) or median. Student's *t* test and Mann-Whitney U test were performed. All the tests were two-sided, *P* < 0.05 was considered to be significant. Statistical analyses were carried out by using SPSS 13.0 software (SPSS, Chicago, IL).
